# National policies and care provision in pregnancy and childbirth for twins in Eastern and Southern Africa: A mixed-methods multi-country study

**DOI:** 10.1371/journal.pmed.1002749

**Published:** 2019-02-19

**Authors:** Claudia Hanson, Stephen Munjanja, Agnes Binagwaho, Bellington Vwalika, Andrea B. Pembe, Elsa Jacinto, George K. Chilinda, Kateri B. Donahoe, Sikolia Z. Wanyonyi, Peter Waiswa, Muchabayiwa F. Gidiri, Lenka Benova

**Affiliations:** 1 Department of Disease Control, London School of Hygiene &Tropical Medicine, London, United Kingdom; 2 Department of Public Health Sciences–Global Health, Karolinska Institutet, Stockholm, Sweden; 3 Department of Obstetrics and Gynaecology, University of Zimbabwe College of Health Sciences, Harare, Zimbabwe; 4 University of Global Health Equity, Kigali, Rwanda; 5 Department of Obstetrics and Gynaecology, University of Zambia School of Medicine, Lusaka, Zambia; 6 Department of Obstetrics and Gynaecology, Muhimbili University of Health and Allied Sciences, Dar-es-Salaam, Tanzania; 7 Women’s and Children’s Health Department, National Directorate for Public Health, Ministry of Health, Maputo, Mozambique; 8 Department of Obstetrics and Gynaecology, Medical College, Blantyre, Malawi; 9 Department of Obstetrics and Gynecology, Aga Khan University, Nairobi, Kenya; 10 College of Health Sciences, Makerere University School of Public Health, Kampala, Uganda; 11 Faculty of Epidemiology and Population Health, London School of Hygiene & Tropical Medicine, London, United Kingdom; 12 Department of Public Health, Institute of Tropical Medicine, Antwerp, Belgium; Cambridge University, UNITED KINGDOM

## Abstract

**Background:**

High-risk pregnancies, such as twin pregnancies, deserve particular attention as mortality is very high in this group. With a view to inform policy and national guidelines development for the Sustainable Development Goals, we reviewed national training materials, guidelines, and policies underpinning the provision of care in relation to twin pregnancies and assessed care provided to twins in 8 Eastern and Southern African countries: Kenya, Malawi, Mozambique, Rwanda, Tanzania, Uganda, Zambia, and Zimbabwe.

**Methods and findings:**

We located policies and guidelines by reviewing national repositories and by contacting experts to systematically map country-level maternal and newborn training materials, guidelines, and policies. We extracted recommendations for care for twins spanning ante-, intra-, and postpartum care that typically should be offered during twin pregnancies and childbirth. We compared care provided for mothers of twins to that provided for mothers of singletons during the ante-, intra-, and postpartum period and computed neonatal mortality rates using the most recent Demographic and Health Surveys (DHS) data for each country. There was a paucity of guidance on care specifically for twin or multiple pregnancies: None of the countries provided clear guidance on additional number of antenatal care visits or specific antenatal content, while 7 of the 8 countries recommended twins to be delivered in a comprehensive emergency obstetric and neonatal care facility. These results were mirrored by DHS results of 73,462 live births (of which 1,360 were twin) indicating that twin pregnancies did not receive more frequent or intensified antenatal care. The percentage of twin deliveries in hospitals varied from 25.3% in Mozambique to 63.0% in Kenya, and women with twin deliveries were between 5 and 27 percentage points more likely to deliver in hospitals compared to women with singleton live births; this difference was significant in 5 of the 8 countries (*t* test *p <* 0.05). The percentage of twin deliveries by cesarean section varied from 9% in Mozambique to 36% in Rwanda. The newborn mortality rate among twins, adjusted for maternal age and parity, was 4.6 to 7.2 times higher for twins compared to singletons in all 8 countries.

**Conclusions:**

Despite the limited sample size and the limited number of clinically relevant services evaluated, our study provided evidence that mothers of twins receive insufficient care and that mortality in twin newborns is very high in Eastern and Southern Africa. Most countries have insufficient guidelines for the care of twins. While our data do not allow us to make a causal link between insufficient guidelines and insufficient care, they call for an assessment and reconceptualisation of policies to reduce the unacceptably high mortality in twins in Eastern and Southern Africa.

## Abstract 2

Claudia Hanson and colleagues review national guidance available in Eastern and Southern African countries for the care of twin pregnancies, and use Demographic and Health Survey data from each country to assess current obstetric practices and outcomes for twins.

## Author summary

**Why was this study done?:**

- Recent publications have shown high mortality in twin pregnancies in low- and middle-income settings.
- Previous research, limited to a few countries in sub-Saharan Africa, suggested that twin pregnancies receive substandard care.
- Very little is known about the underlying reasons for substandard care provision, which constrains the development of specific interventions.
- We sought to assess factors behind substandard care—specifically country policies and guidelines underpinning the provision of care for twins—and the care provided, using the Demographic and Health Surveys data from 8 Eastern and Southern African countries.

**What did the researchers do and find?:**

- We mapped and reviewed national training materials, guidelines, and policies underpinning the provision of care.
- We also assessed antenatal and childbirth care provided to twins based on nationally representative surveys including 1,360 twin births out of 73,462 live births in Kenya, Malawi, Mozambique, Rwanda, Tanzania, Uganda, Zambia, and Zimbabwe.
- Three of 5 countries with national emergency obstetric care in-service training materials omitted a specific section for management of twin deliveries, while university-led pre-service trainings included twins consistently.
- None of the training materials, guidelines, or policies provided clear guidance on an additional number of antenatal visits for twin pregnancies, while 7 of the 8 countries recommended twins be delivered in hospitals providing comprehensive emergency obstetric care.
- The percentage of twin deliveries in hospitals varied from 25.3% in Mozambique to 63.0% in Kenya, and women with twin deliveries were between 5 and 27 percentage points more likely to deliver in hospitals compared to women with singletons.

**What do these findings mean?:**

- These findings suggest that the lack of specific national training materials, guidelines, and policies may be one reason for the suboptimal care provided to twin pregnancies, although our 2 findings of (i) substandard guidelines and (ii) substandard care cannot be linked formally.
- The findings call for more attention within national training materials, guidelines, and policies towards this high-risk group within the Sustainable Development Goal programming.
- Research is needed to investigate whether insufficient diagnosis of twin pregnancies, insufficient clinical guidance, or both critically affect care provision and are important beyond other demand side factors.

## Introduction

Large improvements in child mortality in low- and middle-income settings within the past 25 years have led to greater attention to early life as well as high-risk groups, including multiple gestation pregnancies [1–3]. Pooling data from 30 sub-Saharan African countries, Monden and Smits reported a 5-fold higher neonatal mortality in twins compared to singletons (2009–2014) [3]. Similarly, smaller studies from sub-Saharan Africa reported 3- to 4-fold higher neonatal risk ratios in twins compared to singletons [4–8]. While we lack information from low-income countries, 5-fold higher stillbirth rates are seen in twins compared to singletons in high-income settings [9–11]. Multiple pregnancies are also associated with an increased occurrence of hypertensive disorders, anaemia, and ante- and postpartum bleeding [8,12], resulting in higher rates of potentially life-threatening conditions in mothers of twins compared to mothers of singletons, partially compounded by the fact that twin births are more likely in older mothers [13].

Twin pregnancies exceed 2% of the total births in parts of sub-Saharan Africa [14]. Recent evidence suggests that 10%—or 300,000 deaths—of the 3 million deaths in children below 5 years are in twins in sub-Saharan Africa [3]. About 60% of twins are delivered prematurely [15], necessitating special neonatal care. While twins can be safely delivered vaginally if the leading twin is in a cephalic position [16], cesarean section delivery is indicated if the leading twin is in a breech or transverse position. Typically, delivery in a referral facility with sufficient obstetric and neonatal capacities, including access to cesarean section, is recommended, as well as intensified screening and care for hypertensive disorders, anaemia, premature labour, and fetal growth [17].

While twin pregnancies deserve special care, studies indicate that mothers of twins fail to receive intensified antenatal care (ANC) or to be delivered at a higher rate in facilities compared with singletons [3,18]. Similarly, Virgo et al. suggested that women with pregnancy risk factors in general do not seek care in facilities more often than those without risk factors [19]. ANC services in low-resource settings have struggled to implement the most basic interventions, such as detection and care for anaemia and hypertensive disorders [20–22]. The focused ANC strategy advocated by the World Health Organization (WHO) since the early 2000s puts major emphasis on providing services to all pregnant women regardless of the risk profile [23,24]. The new WHO ANC guidelines, published in 2016 [25], revisited the ANC strategy and risk screening. The guidelines now explicitly recommend early ultrasound for all pregnancies to detect multiples and to determine chorionicity, preferably before 24 weeks where feasible [25].

In view of the increasing importance of focusing on multiple pregnancies for sustained mortality reduction, and in view of the repositioning of risk screening for twins during ANC, we aimed to review present practices for twin pregnancies from the perspective of national recommendations and in terms of care actually received. We reviewed national training materials, guidelines, and policies underpinning the provision of care in relation to twin pregnancies and assessed care provided to twins using data from Demographic and Health Surveys (DHS) in 8 Eastern and Southern African countries: Kenya, Malawi, Mozambique, Rwanda, Tanzania, Uganda, Zambia, and Zimbabwe.

## Methods

The 8 Eastern and Southern African countries were selected based on availability of recent DHS data [26] and collaborators interested in joining the research team to conduct this mixed-methods study.

### Mapping and analysis of training materials and guidelines

We located documents spanning training materials (national and international trainings), national textbooks used in pre- and in-service training, guidelines, and policies by (i) reviewing national repositories, (ii) contacting experts from ministries of health, and (iii) contacting teachers at national training institutions (S1 Fig; S1 Table). National training materials were defined as books, book chapters, or participants’ or trainers’ manuals with reference to antenatal, intrapartum, or postnatal care. Guidelines were defined as any document that aimed to provide an overview on what care is to be provided, including clinical flowcharts. We also considered the antenatal card, the woman-held record documenting care throughout pregnancy and childbirth, as one such document. Policies were included in the final list of documents if they related to clinical or organisational aspects of care provision for maternal and newborn health. The initial research was done in January 2018. Based on the initial search, we compiled a list of country-level training materials, guidelines, and policies relating to maternal and newborn care. We crosschecked the obtained list of training materials, guidelines, and policies with country members of the professional associations of obstetrics and gynaecology as well as midwifery associations, international agencies, and responsible persons of pre-service trainings in obstetrics and gynaecology and midwifery in February and March 2018. Our country co-authors asked professional contacts at the ministries of health and professional organisations for training materials specifically on focused ANC and emergency obstetric care. Saturation was defined when no new documents could be identified by our country co-authors within their professional networks.

Two authors, the country representative and CH, extracted the information on care for twins from located documents using a data extraction form that was piloted and refined to capture all elements mentioned in relation to twin pregnancy and childbirth (S2 Table). The data extraction form included preventive, diagnostic, and curative actions, spanning topics typically covered in obstetric textbooks (identification of twin pregnancies, special care during pregnancy, timing and mode of delivery, special care and precaution during birth, and special care for the newborns including breastfeeding) [17,27] and WHO guidance [28].

### Analysis of care received in pregnancy and childbirth

#### Data

We used the most recent DHS dataset for each included country available as of April 2018. DHS surveys are cross-sectional, nationally representative household surveys. Standard model questionnaires are used but can be adapted by each country. Manuals and technical assistance ensure that the survey procedures followed in each country are similar, providing data comparable across countries. The surveys include questions on household and individual characteristics, child survival and health, and details on antenatal and childbirth care; the questionnaires are administered at the household level by trained interviewers. Data are generally based on the self-report of women of reproductive age (15–49 years) within sampled households. The sampling design is a multi-level cluster survey. Our analytical plan, categories used, and the outline of the tables were agreed on within the team of authors prior to the data analysis in a series of telephone conferences.

#### Population

All women aged 15–49 years with 1 or more live births in the 5-year survey recall period were included in the analysis. We defined a delivery as an event of giving birth; a woman experienced 1 delivery event regardless of whether she delivered a singleton or twins.

#### Analysis

We examined the coverage with ANC by looking at the percentage of women who reported initiating ANC in the first trimester of pregnancy (before month 4 of pregnancy) and who received 4 or more antenatal visits during pregnancy. Content of ANC was assessed by looking at the percentage of women who used ANC receiving each of 5 components measured on surveys: having had blood pressure measured at least once during ANC, having had a urine sample taken, having had a blood sample taken, taking iron supplementation, and receiving information on pregnancy complications. A small percentage (<5%) of women who used ANC reported not knowing/remembering whether they received these care components; we re-coded their responses as not having received this care, as done in previous studies [29].

We assessed the location of the most recent delivery (percentage in any health facility and in a hospital). Hospitals were defined as locations that have the capacity to provide comprehensive emergency obstetric care for women with complications. The categorisation of delivery locations into hospital versus lower level was done in consideration of the local context and health system in each country and the response options on the survey, and is shown in S3 Table. The small number of observations for which type of delivery facility was missing were categorised as lower level. We also estimated the percentage of singleton and twin deliveries by cesarean section. Cesarean sections were only reported for deliveries in health facilities, and the few deliveries where mode of delivery was missing were excluded from analysis of cesarean section. Content of immediate postnatal care for mothers and babies was assessed by examining the percentage of all deliveries where the newborn was weighed after birth and where the newborn was breastfed immediately (within an hour of birth). In the Kenya survey, a random half of the sample of women were not asked about care content; these were excluded from the analysis.

We performed descriptive statistics and assessed differences in relation to socio-demographic characteristics of all singleton and twin deliveries in the 5-year survey recall period. Triplets and higher order multiples were excluded as a rare event—only 14 such deliveries were captured on the 8 surveys used. We assessed differences between singleton and twin live births for maternal age at delivery, parity before the index delivery, household wealth score, and maternal years of education. Women could contribute more than 1 delivery if they had more than 1 live birth in the recall period; their values for maternal age and parity were calculated at the time of each delivery event. However, for coverage and content of ANC and delivery care, we examined only the most recent delivery resulting in a live birth. We also calculated the newborn mortality rate per 1,000 live births.

We hypothesised that women who had twin deliveries, if correctly identified during pregnancy, would have received higher coverage and content of ANC and delivery care. We present *p*-values for *t* tests showing the difference in proportions. For selected indicators across the pregnancy and delivery care continuum, we also provide rate ratios comparing twin to singleton deliveries.

We tabulated missing data and performed a sensitivity analysis for variables that exceeded 3% missing data: We present analyses where missing data are treated in the opposite way as proposed in the main analysis and where missing data are excluded (S1 Text).

Analysis was conducted using Stata 15 SE (StataCorp, College Station, Texas) and adjusting for survey design (*svyset* for clustering, survey weights, and stratification). We consider *p*-values < 0.05 to be statistically significant. Findings from each country are presented separately; no pooled analysis was conducted due to contextual heterogeneity. This study is reported as per the Strengthening the Reporting of Observational Studies in Epidemiology (STROBE) guideline (S1 Checklist).

#### Ethical approval

We used data in the public domain only. The DHS receive government permission, use informed consent of the head of household and interviewed women, and assure respondents of confidentiality. The Research Ethics Committee of the London School of Hygiene & Tropical Medicine approved our analyses.

## Results

### Training materials, guidelines, and policies

We identified 62 training materials, guidelines, and policies (S4 Table) with varying availability and focus between the countries. For example, ANC training guidelines were only available in Rwanda and Tanzania. In 3 countries (Malawi, Zambia, and Zimbabwe), obstetricians and midwives had prepared training materials for university-led pre-service training while in Tanzania, Mozambique, Uganda, Rwanda, and Kenya, important training materials (life-saving skills or emergency obstetric care trainings) were prepared by the ministry of health. In addition, international training curricula and materials were reviewed, such as the Advances in Labour and Risk Management (ALARM) course manual [30], the Advanced Life Support in Obstetrics (ALSO) [31], and the life-saving skills *Making It Happen* training [32]. These were implemented to varying degrees in Uganda, Kenya, Tanzania, Zimbabwe, Malawi, and Rwanda.

In 7 out of the 8 countries, at least 1 document highlighted twin pregnancies as being at increased risk, with particular reference to complications such as anaemia and pre-eclampsia (Table 1).

**Table 1:** Guidance from training materials, guidelines, and policies on identification and care for multiple pregnancies and babies born with their sibling.

Subject	Country							
	Kenya	Malawi	Mozambique	Rwanda	Tanzania	Uganda	Zambia	Zimbabwe
Twin pregnancy highlighted as risk factor for maternal and perinatal mortality	Risk factor for pre-eclampsia/eclampsia^1^; can be the cause of bleeding in pregnancy/threatened abortion^1^	Risk factor for anaemia, hyperemesis gravidarum, hypertensive disorders, APH, thromboembolism, preterm labour, prolonged labour, and PPH^2^		Risk factor for anaemia^3^, APH^4^, and fetal growth restriction^4^	Risk factor for PPH^5^ and premature birth^6^	High-risk pregnancy^7^; risk factor for PPH^7^	Risk factor for miscarriage, APH, hypertensive disorders, PPH, stillbirths, and neonatal death^8^^,^^9^	Risk factor for anaemia, hypertensive disorders, prematurity, LBW, APH, PPH, and mortality^10^^,^^11^
Identification of twin pregnancies during ANC	Mismatch in fundal height and gestational age, exaggerated pregnancy symptoms, multiple fetal parts^1^; family history of twins or previous twin pregnancy assessed during history taking^1^^,^^12^	Mismatch in fundal height and gestational age, exaggerated pregnancy symptoms^2^; additional ANC visits^2^; to be checked by scan for chorionicity^2^	Mismatch in fundal height and gestational age or increase in weight gain of more than 2 kg^13^	Mismatch in fundal height and gestational age, 2 audible fetal heartbeats, multiple fetal parts, exaggerated symptoms of pregnancy^4^	Mismatch in fundal height and gestational age^5^; excessive weight gain^14^	Family history of twins^15^^,^; exclude multiple pregnancy (unspecific advice)^7^; hyperemesis might be sign for multiples, to be checked by scan^7^	Exaggerated symptoms of pregnancy, family history, multiple fetal parts^8^^,^^9^; use scan^8^^,^^9^	Mismatch in fundal height and gestational age, difficult palpation/several poles^11^; family history of twins^11^; use scan before 24 weeks to diagnose twin pregnancy^10^
Special care during pregnancy	Admission to facility in case of pregnancy complication such as APH, preterm labour^1^; higher weight increase recommended^1^	Additional caloric need^2^; scan every 2–3 weeks^2^; corticosteroids at 28 weeks for monoamniotic pregnancies^2^		Check for anaemia^3^^,^^16^; can be the cause of threatened abortion^3^^,^^16^; monitor for associated obstetric complications, determine presentation, perform ultrasound^4^	Manage multiple pregnancies (no details)^14^; look out for pre-eclampsia/eclampsia^5^	Higher weight increase recommended^17^	Ultrasound for dating and chorionicity and growth discordance, monthly (later biweekly) ANC visits, corticosteroids at 26 weeks of gestational age^8^; routine ANC unless there are complications^9^; increased caloric intake^8^^,^^9^	Check for anaemia and urine for protein, watch blood pressure very closely, 3 scans, plan mode of delivery^11^
Advice on place of delivery	Comprehensive emergency obstetric care facility^1^	In case of discordant growth or monoamniotic pregnancy, refer to Central Hospital^2^	Comprehensive emergency obstetric care facility^13^	Hospital^4^	Hospital^18^		Hospital^9^	Specialised care (no other specifics)^19^; intrauterine fetal death of 1 twin is indication for referral to tertiary institution^11^
Advice on timing of delivery		No later than 39 weeks, monoamniotic pregnancy between 32–34 weeks of gestation^2^					Consider; CS at 36 weeks of gestation if monoamniotic pregnancy^8^; plan delivery no later than 40 weeks^8^; induction of labour at 38 weeks of gestation^9^	
Advice on mode of delivery	Risk factor for obstructed labour^1^; consider CS if non-cephalic first twin, retained second twin, extreme prematurity, discordant or monozygotic twins, and >2 multiples^1^^,^^20^	Vaginal delivery if first twin in cephalic presentation and no complication^2^		Consider CS if first twin not cephalic, previous scar, and >2 fetuses^4^			Consider CS if non-cephalic first twin or >2 multiples^8^; low threshold for CS^8^	Labour is managed according to the presentation of the first twin, active management of delivery of second twin, watch out for fetal distress^11^
Care during birth	Management of twin delivery part of a distinct chapter^20^	Obstetric and paediatric registrars need to be present, prepare 2 delivery sets^2^; risk factor for resuscitation^21^	Check for second twin before AMTSL^22^	Risk factor for PPH^16^, deep vein thrombosis^4^, and pulmonary embolus^4^	Risk factor for PPH^5^; rule out second twin before AMTSL^14^^,^^23^	First twin at greater risk of HIV^7^; risk factor for obstructed labour^7^; exclude second baby after delivery^7^	Registrar needs to be present for delivery, prepare 2 delivery sets, watch for intrauterine fetal death^8^; obstetrician and paediatrician should be present, active management of delivery of second twin, check very carefully for PPH^9^	Registrar needs to be present for delivery^11^
Advice on breastfeeding					Detailed guidance on how to support breastfeeding in twins^24^^,^^25^			
Postnatal care/advice on special neonatal care	Mentioned as risk factor^1^^,^^20^		Mentioned as risk factor on child health card^26^		Mentioned as risk factor on child health card^27^	Special care and more frequent follow-up needed, keep longer in facility^7^		

^1^Kenya Ministry of Public Health and Sanitation and Kenya Ministry of Medical Services. National Guidelines for Quality Obstetrics and Perinatal Care.

^2^Association of Obstetricians and Gynaecologists of Malawi. Obstetrics and Gynaecology Protocols and Guidelines. Version 3.0. April 2017.

^3^Rwanda Ministry of Health. Focused Antenatal Care. Reference Manual for Health Care Providers. 2014.

^4^Rwanda Ministry of Health. Gynecology and Obstetrics. Clinical Protocols & Treatment Guidelines. 2012.

^5^Tanzania Ministry of Health and Social Welfare. Learning Resource Package for Basic Emergency Obstetric and Newborn Care (BEmONC). Participant’s Handbook. 2010.

^6^Tanzania Ministry of Health and Social Welfare. Administration of Antenatal Corticosteroids in Pre-Term Labour. 2015.

^7^Uganda Ministry of Health. Uganda Clinical Guidelines. National Guidelines for Management of Common Conditions. 2016.

^8^University of Zambia Medical Education Partnership Initiative. Obstetrics & Gynecology Protocols and Guidelines. 2014.

^9^Lusaka School of Midwivery, Lecturer Mr Sikuyuba. Lesson Plan, Obstetrics and Midwifery Care, Multiple Pregnancies. 2017.

^10^Zimbabwe Ministry of Health and Child Welfare. National Guidelines on Key Interventions to Improve Perinatal and Neonatal Health Outcomes in Zimbabwe. 2017.

^11^University of Zimbabwe Department of Obstetrics and Gynaecology. Essential Guide to Management of Common Obstetric and Gynaecologic Conditions in Zimbabwe. 2012.

^12^Kenya Ministry of Health. Maternal & Child Health Booklet. Afya Ya Mama Na Mtoto.

^13^Mozambique Ministry of Health. Caderneta de Saúde da Mulher. 2015.

^14^Tanzania Ministry of Health and Social Welfare. Focused Antenatal Care. Malaria and Syphilis during Pregnancy. Learner’s Guide for ANC Service Providers and Supervisors. 2009.

^15^Uganda Ministry of Health. Ante-Natal Card.

^16^Rwanda Ministry of Health. Continuous Training in Emergency Basic and Comprehensive Obstetric and Neonatal Care. Reference Manual. 2015.

^17^Uganda Ministry of Health. Guidelines on Maternal Nutrition in Uganda. 1st edition. 2010.

^18^Tanzania Ministry of Health and Social Welfare. Kadi Ya Kliniki Ya Waja Wazito (Antenatal Card) RCH4. 2006.

^19^Zimbabwe Ministry of Health. Maternal and Neonatal Health Record.

^20^Kenya Ministry of Public Health & Sanitation. Emergency Obstetric and Neonatal Care Guidelines. A Harmonized Competency Based Training Curriculum for Kenya.

^21^Paediatrics and Child Health Association. Care of the infant and newborn in Malawi. The COIN Course. Participants Manual.

^22^Mozambique Ministry of Health. Padrões para Medição do Desempenho dos Serviços de Saúde Materna e Neonatal. 2016.

^23^Tanzania Ministry of Health and Social Welfare. Active Management of the Third Stage of Labor (AMTSL).

^24^Tanzania Ministry of Health and Social Welfare. Kangaroo Mother Care Guideline. 2008.

^25^Tanzania Ministry of Health and Social Welfare. Infant and Young Child Feeding. July 2013.

^26^Mozambique Ministry of Health. Cartáo de Saúde da Crianca.

^27^Tanzania Ministry of Health and Social Welfare. Kadi Ya Kliniki Ya Mtoto RCH 1.

AMTSL, active management of the third stage of labour; ANC, antenatal care; APH, antepartum haemorrhage; CS, cesarean section; LBW, low birth weight; PPH, postpartum haemorrhage.

Advice on identification of twin pregnancy referred to exaggerated symptoms of pregnancies such as “increased symptoms of early pregnancy (i.e. nausea, vomiting)” [33] or a mismatch between gestational age and fundal height, which is to be checked by an ultrasound scan. Only training materials from Zimbabwe proposed a scan before 24 weeks as a primary diagnostic measure for all pregnancies. In none of the countries was explicit guidance found on additional ANC visits for confirmed twin pregnancy. Documents in all countries, except in Uganda, explicitly recommended that twin deliveries should be planned in a hospital or a comprehensive emergency obstetric care facility, in other words a facility able to perform a cesarean section. In Kenya, for example, the national guideline on quality obstetric and neonatal care recommended “in case of suspected twins, advise mother to visit a facility that can provide Comprehensive Emergency Obstetric and Newborn Care to prepare for delivery” [34]. In Mozambique and Tanzania, such advice was, however, only available as a note on the antenatal card. Special advice on the mode of delivery was only available in half of the countries (Kenya, Malawi, Rwanda, and Zimbabwe). Life-saving skills and emergency obstetric care trainings gave very limited advice on how to manage a twin delivery, with no advice on care during birth in Rwanda, Tanzania, and Uganda. The training materials in Kenya and Zambia included a short session on twin deliveries, but no details were available on the content. Thus it remains unclear if guidance included advice on mode of delivery, management of the second twin, and precautions to be taken, such calling a second paediatrician for standby. Locally produced textbooks and training materials prepared by obstetricians and midwives to support university-led pre-service training included more details on twin pregnancies and deliveries, including advice on specialist care, assessing chorionicity using ultrasound for risk determination, preparation of double delivery sets, and sufficient staff. Only kangaroo mother care and infant and young child feeding guidelines in Tanzania included advice on breastfeeding twins. The 3 international trainings of ALARM, ALSO, and *Making It Happen* included a dedicated chapter for twin pregnancy and childbirth (S4 Table). The Jhpiego-promoted trainings of Helping Babies Breathe and Helping Mothers Survive did not include any special aspects of twin care.

### Care received in pregnancy and childbirth

The dates of the 8 included DHS surveys ranged from 2011 (Mozambique) to 2016 (Uganda). The sample of women with live births in the 5-year recall period ranged from 4,832 in Zimbabwe to 14,949 in Kenya (Table 2), with a total of 100,829 live birth deliveries. The percentage of live birth deliveries that resulted in multiple births (twins and higher) ranged from to 1.42% in Rwanda to 2.07% in Mozambique; the vast majority of multiple births were twins. Twins were therefore a relatively rare event, but of the 8 included surveys, there was a sample of >100 such deliveries in each country in the 5-year recall period, a total of 1,695 in the 8 countries. Compared to women who had singleton deliveries, women who had twins were significantly (*p <* 0.05) older in every country except in Rwanda. Across the 8 countries, the average difference in mean maternal age of women delivering twins versus singletons was 1.9 years (largest in Zambia, 3.4 years). Similarly, women with live twin deliveries were of significantly higher parity compared to women delivering singletons in all countries except for Rwanda. The average extent of this difference was 0.5 years across the countries. We found no meaningful household wealth and education level differences between women having singletons and women having twin deliveries (S6 Table), although we detected a statistically significant difference in wealth in Rwanda (women having twins were poorer) and in education in Kenya (mothers of twins had slightly more years of education) and in Zambia (mothers of twins had slightly fewer years of education). The newborn mortality rate for twins ranged from 92.6 per 1,000 live births in Rwanda to 148.2 in Malawi, and in every country was significantly higher than the rate among singletons.

**Table 2:** Characteristics of all deliveries resulting in live births in the survey recall period, by country.

Country	Survey year	Sample size		Percent among deliveries resulting in live births			Maternal age at delivery: mean (95% CI)			Parity before index delivery: mean (95% CI)			Number live born		Newborn mortality rate per 1,000 live births (<30 days)*	
		Women with live births	Live birth deliveries	Singleton	Twins	Triplets and higher	Singleton	Twins	*p*-Value	Singleton	Twins	*p*-Value	Singletons	Twins	Singletons	Twins
Kenya	2014	14,949	20,672	98.59%	1.43%	0.00%	26.5 (26.3–26.6)	27.7 (26.7–28.7)	<0.001	2.2 (2.1–2.2)	2.5 (2.1–2.9)	<0.001	20,380	584	18.8	116.2
Malawi	2015–2016	13,448	16,951	98.04%	1.99%	0.01%	25.9 (25.8–26.1)	27.6 (26.8–28.4)	<0.001	2.2 (2.1–2.2)	2.6 (2.4–2.9)	<0.001	16,618	662	21.5	148.2
Mozambique	2011	7,622	10,879	97.96%	2.07%	0.01%	26.6 (26.3–26.8)	28.3 (27.2–29.5)	<0.001	2.5 (2.4–2.6)	3.0 (2.6–3.3)	<0.001	10,657	442	26.5	143.0
Rwanda	2015	5,955	7,742	98.57%	1.42%	0.04%	28.4 (28.2–28.6)	29.1 (28.0–30.2)	0.299	2.0 (1.9–2.1)	1.9 (1.5–2.3)	0.624	7,631	216	17.8	92.6
Tanzania	2015–2016	7,050	10,048	98.18%	1.83%	0.02%	27.0 (26.7–27.2)	29.8 (28.7–31.0)	<0.001	2.5 (2.4–2.6)	3.2 (2.7–3.6)	0.021	9,865	362	22.1	109.8
Uganda	2016	10,262	15,266	98.34%	1.68%	0.01%	26.4 (26.2–26.6)	27.3 (26.4–28.2)	0.013	2.7 (2.7–2.8)	3.0 (2.6–2.5)	0.026	15,012	504	23.3	111.8
Zambia	2013–2014	9,350	13,244	98.41%	1.59%	0.02%	26.7 (26.5–26.9)	30.1 (29.0–31.2)	<0.001	2.7 (2.6–2.8)	3.7 (3.3–4.1)	<0.001	13,034	414	20.8	116.2
Zimbabwe	2015	4,832	6,027	98.27%	1.74%	0.02%	26.5 (26.3–26.7)	29.3 (28.2–30.5)	<0.001	1.8 (1.7–1.9)	2.3 (1.9–2.6)	0.005	5,923	206	25.6	114.8

Multiple births (twins, triplets) are counted as 1 delivery.

*p-Value for difference in newborn mortality between singletons and twins was <0.001 in all included countries.

The analysis samples for coverage and content of ANC and delivery care were restricted to women’s most recent live birth (total of 73,462 live births, of which 1,360 were twin). Table 3 shows that the percentage of women starting ANC in the first trimester of pregnancy was low, exceeding 50% only in Rwanda. There were no meaningful differences in the coverage of early ANC across the countries, and coverage was significantly higher for twins compared to singletons only in Malawi. Similarly, there were no significant differences in coverage with 4 or more ANC visits during pregnancy between singletons and twins in any country. In terms of content of ANC among women who accessed ANC, we found that the percentage of women receiving the 5 assessed care components varied widely across the included countries and care components. However, significant differences in the likelihood of receiving these components between singletons and twins were an exception. Specifically, the percentage of women reporting having had their blood pressure measured at least once during ANC was significantly higher, by around 10 percentage points, among women delivering twins in Rwanda and Uganda. Women with twin deliveries were not significantly more likely to report giving a urine sample or taking iron supplementation compared to women with singletons, in any country. Only 4 of the 8 included country surveys asked women whether they received counselling on potential complications during pregnancy; there were no significant differences between women with twin versus singleton deliveries in these countries. The percentage of women reporting having had a blood sample taken at least once during ANC showed the highest coverage across the 5 care components included in the analysis, but there were no significant differences between women with twins and singletons, except in Zambia, where women with twins were *less* likely to report receiving this care component.

**Table 3:** ANC coverage and content for most recent live birth deliveries in the survey recall period, by country.

Country	Sample size of deliveries		Percent started ANC in first trimester of pregnancy			Percent received 4+ ANC visits			ANC content—among ANC users														
									Percent had blood pressure measured at least once during ANC			Percent had urine sample taken at least once during ANC			Percent had blood sample taken at least once during ANC			Percent took iron supplementation during pregnancy			Percent received information about complications during ANC		
	Singleton	Twin	Singleton	Twin	*p*-Value	Singleton	Twin	*p*-Value	Singleton	Twin	*p*-Value	Singleton	Twin	*p*-Value	Singleton	Twin	*p*-Value	Singleton	Twin	*p*-Value	Singleton	Twin	*p*-Value
Kenya*	14,713	236	19.7%	27.0%	0.055	57.5%	62.4%	0.254	93.9%	96.2%	0.348	88.7%	92.6%	0.290	96.0%	96.8%	0.663	71.9%	66.7%	0.333	58.3%	67.4%	0.119
Malawi	13,161	285	23.9%	29.8%	0.038	50.5%	55.4%	0.169	83.2%	80.7%	0.361	32.2%	33.5%	0.727	92.4%	94.0%	0.379	90.3%	89.7%	0.814	Not measured		
Mozambique	7,455	167	13.1%	15.8%	0.353	50.5%	57.6%	0.136	58.7%	65.7%	0.158	49.5%	57.4%	0.104	84.4%	89.0%	0.179	87.9%	87.2%	0.861	39.5%	43.2%	0.418
Rwanda	5,866	86	56.1%	57.0%	0.884	43.9%	47.0%	0.575	83.9%	93.8%	0.016	58.2%	61.6%	0.527	96.7%	98.9%	0.251	80.1%	87.6%	0.086	78.3%	82.7%	0.338
Tanzania	6,912	137	24.3%	32.5%	0.059	50.6%	52.9%	0.658	70.8%	70.5%	0.951	60.0%	60.4%	0.941	86.9%	88.7%	0.574	82.0%	87.9%	0.124	Not measured		
Uganda	10,064	198	29.1%	29.5%	0.919	59.9%	59.9%	0.995	71.6%	79.6%	0.030	39.3%	46.8%	0.060	93.0%	93.2%	0.939	89.7%	91.6%	0.455	Not measured		
Zambia	9,184	166	24.4%	25.5%	0.782	55.4%	61.0%	0.202	88.9%	88.3%	0.828	41.6%	34.0%	0.103	94.0%	88.7%	0.011	96.6%	98.1%	0.296	87.9%	90.2%	0.414
Zimbabwe	4,747	85	38.5%	36.3%	0.701	75.8%	74.3%	0.797	96.9%	97.1%	0.927	68.0%	76.6%	0.178	98.3%	99.2%	0.448	88.4%	91.5%	0.424	Not measured		

*Kenya content of care indicators were only asked on a random subsample of the respondents, the sample sizes of singleton and twin deliveries were 7,056 and 111, respectively.

ANC, antenatal care.

In regard to delivery care, Table 4 shows that the percentage of singleton deliveries occurring in health facilities ranged from 61.0% in Mozambique to >90% in Malawi and Rwanda. Women with twin deliveries in Kenya, Rwanda, and Zimbabwe were significantly more likely to deliver in health facilities than women with singletons. However, between 2% (Rwanda) and >30% (Mozambique and Tanzania) of twins were born outside of health facilities across the countries. The percentage of twin deliveries in hospitals varied from 25.3% in Mozambique to 63.0% in Kenya. Women with twin deliveries were between 5 and 27 percentage points more likely to deliver in hospitals; this difference was significant in 5 of the 8 countries. The percentage of twin deliveries by cesarean section ranged from 8.6% in Mozambique to 36.3% Rwanda, and was significantly higher than cesarean section rate for singleton deliveries in all countries. The percentage of newborns weighed at birth ranged from around two-thirds to more than 97%, but we found no significant differences between singletons and twins in any country. The sensitivity analysis for Malawi, where more than 6% of responses were missing, gave similar results when missing data were excluded from the analysis, while a lower percentage of weighing was seen in twins when missing data were treated as “not weighed” (S1 Text). The percentage of singleton newborns put to breast within an hour of delivery ranged from around half to four-fifths. In all countries, twins were less likely to initiate breastfeeding immediately—the average difference across the 8 countries was 11 percentage points. This difference was statistically significant in 5 countries. The sensitivity analysis done on data from Mozambique indicated similar differences when missing data were treated as “babies breast fed immediately” or when missing data were excluded from the analysis, but the differences were no longer significant.

**Table 4:** Delivery and postnatal care coverage and content for women’s most recent live birth delivery in the survey recall period, by country.

Country	Delivery location						Mode of delivery			Newborn/postnatal care and practices					
	Percent of all deliveries in a health facility			Percent of all deliveries in a hospital			Percent of all deliveries by cesarean section			Percent of all newborns weighed at birth			Percent of all newborns breastfed within 1 hour of delivery		
	Singleton	Twin	*p*-Value	Singleton	Twin	*p*-Value	Singleton	Twin	*p*-Value	Singleton	Twin	*p*-Value	Singleton	Twin	*p*-Value
Kenya*	67.1%	80.5%	<0.001	48.0%	63.0%	<0.001	9.4%	25.2%	<0.001	71.5%	76.2%	0.385	62.3%	53.0%	0.103
Malawi	93.7%	92.3%	0.367	36.0%	47.5%	<0.001	6.1%	19.4%	<0.001	92.5%	90.1%	0.210	76.5%	65.4%	<0.001
Mozambique	61.0%	65.4%	0.335	20.7%	25.3%	0.190	4.4%	8.6%	0.005	60.5%	62.7%	0.641	75.9%	67.4%	0.038
Rwanda	92.2%	97.9%	0.042	27.2%	54.3%	<0.001	12.7%	36.3%	<0.001	93.6%	97.8%	0.101	79.3%	55.4%	<0.001
Tanzania	67.9%	66.7%	0.792	35.3%	43.6%	0.109	6.7%	21.5%	<0.001	68.4%	68.7%	0.948	52.0%	42.4%	0.088
Uganda	76.7%	75.5%	0.716	37.3%	42.3%	0.179	7.2%	11.9%	0.028	71.3%	67.2%	0.220	65.8%	54.9%	0.004
Zambia	72.9%	79.5%	0.109	24.9%	37.7%	0.001	5.1%	10.5%	0.009	73.7%	76.1%	0.575	65.1%	64.9%	0.962
Zimbabwe	82.1%	92.9%	0.025	40.0%	55.5%	0.014	5.8%	23.4%	<0.001	85.5%	93.8%	0.025	57.3%	44.2%	0.025

Sample sizes as reported in Table 2.

*Kenya content of care indicators were only asked for a random subsample of the respondents; the sample sizes of singleton and twin deliveries were 7,056 and 111, respectively.

Table 5 summarises the differences between singleton and twin deliveries across the ANC and delivery care continuum, culminating in newborn survival. It shows that twin deliveries were, with some exceptions, not more likely to receive early ANC or 4+ ANC visits. However, they were between 1.1 and 2.0 times more likely to occur in a hospital, and 1.7 to 4.0 times more likely to be by cesarean section. Across the included countries, the newborn mortality rate was 4.5 to 6.9 times higher for twins compared to singletons.

**Table 5:** Rate ratios of selected indicators along the continuum of care comparing twin to singleton deliveries (based on results shown in Tables 2–4), by country.

Country	Antenatal care		Delivery care		Survival	
	Started ANC in first trimester	Received 4+ ANC visits	Delivered in a hospital	Delivered by cesarean section	Newborn mortality rate (crude)	Newborn mortality rate (adjusted)
Kenya	1.4	1.1	**1.3**	**2.7**	**6.2**	**6.3**
Malawi	**1.2**	1.1	**1.3**	**3.2**	**6.9**	**7.2**
Mozambique	1.2	1.1	1.2	**2.0**	**5.4**	**5.7**
Rwanda	1.0	1.1	**2.0**	**2.9**	**5.2**	**5.4**
Tanzania	1.3	1.0	1.2	**3.2**	**5.0**	**5.1**
Uganda	1.0	1.0	1.1	**1.7**	**4.8**	**4.7**
Zambia	1.0	1.1	**1.5**	**2.1**	**5.6**	**6.3**
Zimbabwe	0.9	1.0	**1.4**	**4.0**	**4.5**	**4.6**

Rate ratios with *p*-values < 0.05 are shown in bold; mortality rate adjustment included maternal age and parity.

ANC, antenatal care.

## Discussion

The reviewed training materials, guidelines, and policies indicated limited guidance in regard to twin or multiple pregnancies in 8 Eastern and Southern African countries: Recommendations to diagnose twin or multiple pregnancies were based on unspecific symptoms, such as aggravated symptoms of pregnancy. None of the countries provided clear guidance on additional care and number of ANC visits. In 7 of the 8 countries, at least 1 document recommended twins to be delivered in a hospital or comprehensive emergency obstetric and neonatal care facility. Only 2 of 5 identified emergency obstetric and neonatal care training materials included a session on management of twin or multiple delivery. The deficits in guidance and advice were mirrored by the observed care received: Twin pregnancies did not receive earlier, meaningfully more frequent, or intensified ANC. Still, women with twin deliveries were between 5 and 27 percentage points more likely to deliver in hospitals; this difference was significant in 5 of the 8 countries. The percentage of twin deliveries by cesarean section varied from 9% in Mozambique to 36% Rwanda. Newborn mortality rate was 4.5 to 6.9 times higher for twins compared to singletons in the 8 countries.

It has also been raised in other studies twin pregnancies do not receive sufficient attention and care during the ante- and intrapartum period. In Nigeria, the average number of ANC visits was similar for singleton and multiple pregnancies, while more women of multiples delivered with a skilled attendant [18]. Bellizzi et al., pooling all DHS data from 60 low- and middle-income countries, also reported only a marginally increased number of ANC visits in twins compared to singletons [35]. However, since the rate of premature delivery is higher in twins, they are less likely to be exposed to the same number of visits, ANC procedures, and investigations, which might partially explain the results.

We observed low rates of cesarean section among twin deliveries: between 9% (Mozambique) and 36% (Rwanda). Very low cesarean section rates in twin deliveries were also reported from Nigeria, at only 7% [18]. This is in contrast to studies in high- and higher-middle-income countries, where 60%–70% of deliveries in multiples are reported to be by cesarean section (63% in the UK, [36] 60% in Peru [37], 70% in Singapore [38], and 68% in Thailand [39]). The low rate of cesarean section might reflect the cascade of missed opportunities during ANC, insufficient referral advice for hospital care, missing clinical skills to manage twin deliveries, and, to a lesser degree, methodological issues, as we missed stillbirth deliveries.

Of particular concern is also the fact that there was still a substantial fraction of twin deliveries at home (more than 1 in 5 deliveries in 5 of the 8 countries included). Our data cannot provide any explanation for why women delivered at home as we have no information about whether the twin pregnancy was not diagnosed or the women experienced fast premature labour or faced accessibility problems for other reasons. We report newborn mortality rates 5- to 7-fold higher in twins compared to singleton pregnancies, which is in line with what Monden and Smits reported based on DHS studies from 2009–2014 in sub-Saharan Africa [3]. Again, we are missing information about the extent to which too late referral, inadequate management, or other complicating diseases such as hypertensive disorder contribute to mortality.

Our findings confirm that risk screening and management of high-risk pregnancies is insufficient in Eastern and Southern Africa, a concern also raised by Virgo et al. [19]. Surprisingly, training materials on ANC were not consistently available in the 8 countries, and often only the ANC card provided advice on where women with risk factors should deliver. It is worth noting that the focused ANC training materials from Tanzania and Rwanda were silent on advice for screening and management of twin or multiple pregnancies.

The lack of policy and recommendations for identification of twin pregnancies might be explained by the policy shift in ANC in the early 2000s from a focus on risk identification to service delivery, e.g., malaria prophylaxis [40,41]. This shift was further supported by the slogan “Every pregnancy faces risk” [42] and the inclusion of the skilled birth attendant indicator in the Millennium Development Goals.

We believe this shift might have resulted in too little attention on triaging and referral of high-risk pregnancies. While the risk factors age, parity, and height are little predictive of pregnancy complications, a twin pregnancy carries an important increased risk, and complications need to be prevented.

Still, risk detection alone without improved referral and management of high-risk pregnancies will not improve outcomes, as the first trial on ultrasound screening in low- and middle-income settings suggests [43]. However, the high mortality in twins and multiples makes it paramount to give greater attention to this high-risk group within the Sustainable Development Goals and the Every Women, Every Child agendas [44,45]. Targeted country policies and guidelines, as our study indicates, need to support twin pregnancies, from identification including ultrasound to referral, appropriate management, and special care after birth.

### Methodological considerations

The strength of our analysis lies in the comprehensiveness of our analysis, spanning from (i) examining training, guidelines and national policies underpinning the provision of care to (ii) care and services provided and (iii) mortality outcomes in 8 Eastern and Southern African countries. By this comprehensiveness, our analysis also provides potential underlying reasons for missed opportunities and high mortality. We used DHS data, which are nationally representative; thus, results are generalisable to the respective country. The dataset was very complete, with few missing data.

Still, our analysis faced limitations. We used women’s reports of care received during pregnancy and childbirth, which are prone to recall bias, and we cannot exclude some errors, for example, misclassification of newborn deaths as stillbirths [46]. It is also likely that we have missed some twin deliveries where 1 twin was born alive and the other stillborn—these might have been misreported as singleton live births. Stigmatisation of twin deliveries in some societies might have resulted in underreporting of twin live births. Moreover, there are reports that women have problems in accurately recalling certain interventions, such as immediate breastfeeding [47], and it is known that women who undergo cesarean section may miss recognising that their baby’s weight was taken. Also, the sample of twin deliveries was small, especially in the sample restricted to women’s most recent live delivery, for which information on ANC is available. Thus, we faced limited power to detect differences. Still, the sample was large enough for clinically meaningful differences of about 10 percentage points and wider. Finally, we were lacking information on mode of conception (in vitro fertilisation or not) and chorionicity, which is a key determinant of perinatal outcomes in twin pregnancies. We also cannot determine whether the lack of detection of twin pregnancies or the missing guidance on the needed intensified care in pregnancy and childbirth resulted in the insufficient care. However, we believe that both aspects are interlinked and that only an experimental study could shed light on this question.

We faced limitations in categorising hospitals and referral delivery facilities. Some DHS surveys include more answer options, others less. While we grouped the available categories into hospital and lower level on advice of our national collaborators, we cannot exclude some misclassification due to survey response options or women’s recall.

We cannot exclude that we missed training materials, guidelines, and policies advising on care for twins. However, our country collaborators were obstetricians or maternal newborn health researchers with excellent professional networks in their countries, and they were confident that they identified all documents.

### Conclusion

Our analysis of the most recent nationally representative household surveys collecting data on live singleton and twin births indicated important missed opportunities in providing special or intensified care to mothers of twins. Most countries’ guidelines had insufficient specific advice and recommendations for the care of twin pregnancies. While our data do not allow us to make a causal link between these 2 findings, it is plausible that one of the reasons for substandard care is the lack of guidance. However, the lack of identification of twin pregnancies might also explain results, and both the lack of identification and insufficient guidance are probably interlinked.

We recommend that, in view of the policy window created by the new WHO ANC and intrapartum guidelines, high-risk pregnancies like multiple pregnancies should receive increased attention in policies, guidelines, and trainings to identify risks as well as to define special and intensified care. While the slogan “Every pregnancy faces risk” was important to encourage mothers to deliver in a facility, we might need to add “Some mothers and babies face much greater risks”.

Our study also calls for further research: For policies and guidelines to be specific and relevant, we need to characterise the key bottlenecks and to design and evaluate interventions that address identification of twin deliveries, referral, appropriate management, and care.

## Supporting information

S1 Checklist STROBE checklist. (DOC)

S1 Fig Flowchart of mapping. (TIF)

S1 Text Sensitivity analysis of missing data in the DHS surveys. (DOCX)

S1 Table Ministry of health addresses and agencies. (DOCX)

S2 Table Data extraction form. (DOCX)

S3 Table Response options for facility-based delivery locations. (DOCX)

S4 Table International training details. (XLSX)

S5 Table International training programmes. (DOCX)

S6 Table Deliveries resulting in live births in the survey recall period. (DOCX)

## Abbreviations

ALARM: Advances in Labour and Risk Management
ALSO: Advanced Life Support in Obstetrics
ANC: antenatal care
DHS: Demographic and Health Surveys
WHO: World Health Organization
