# Supplementary material for: National policies and care provision in pregnancy and childbirth for twins in Eastern and Southern Africa: A mixed-methods multi-country study
Source: PLoS Med. 2019 Feb 19;16(2):e1002749. doi: 10.1371/journal.pmed.1002749 (PMC6380547; doi:10.1371/journal.pmed.1002749)
Supplement: S1 Checklist — (DOC) [file pmed.1002749.s001.doc]

**S1 Checklist: STROBE S**tatement

|  | Item No | Recommendation | Section and paragraph number (P) |
| --- | --- | --- | --- |
| **Title and abstract** | 1 | (*a*) Indicate the study’s design with a commonly used term in the title or the abstract | Abstract (P2) |
|  |  | (*b*) Provide in the abstract an informative and balanced summary of what was done and what was found | Abstract (P2-3) |
| Introduction | | |  |
| Background/rationale | 2 | Explain the scientific background and rationale for the investigation being reported | Introduction (P2-3) |
| Objectives | 3 | State specific objectives, including any prespecified hypotheses | Introduction (P4) |
| Methods | | |  |
| Study design | 4 | Present key elements of study design early in the paper | Methods (P1, P4) |
| Setting | 5 | Describe the setting, locations, and relevant dates | Methods (P4) |
| Participants | 6 | (*a*) Give the eligibility criteria, and the sources and methods of selection of participants | Methods (P5) |
| Variables | 7 | Clearly define all outcomes, exposures, predictors, potential confounders, and effect modifiers. | Methods (P6-11) |
| Data sources/ measurement | 8* | For each variable of interest, give sources of data and details of methods of assessment (measurement). Describe comparability of assessment methods if there is more than one group | Methods (P6-8) |
| Bias | 9 | Describe any efforts to address potential sources of bias | Methods (P9-10) |
| Study size | 10 | Explain how the study size was arrived at | Methods (P5), Results (P3) |
| Quantitative variables | 11 | Explain how quantitative variables were handled in the analyses. If applicable, describe which groupings were chosen and why | Methods (P6-8) |
| Statistical methods | 12 | (*a*) Describe all statistical methods, including those used to control for confounding | Methods (P8-11) |
|  |  | (*b*) Describe any methods used to examine subgroups and interactions | N/A |
|  |  | (*c*) Explain how missing data were addressed | Methods (P10) |
|  |  | (*d*) If applicable, describe analytical methods taking account of sampling strategy | Methods (P11) |
|  |  | (*e*) Describe any sensitivity analyses | Methods (P10) |
| Results | | |  |
| Participants | 13* | (a) Report numbers of individuals at each stage of study—eg numbers potentially eligible, examined for eligibility, confirmed eligible, included in the study, completing follow-up, and analysed | Results (P3)  Table 2 |
|  |  | (b) Give reasons for non-participation at each stage | N/A |
|  |  | (c) Consider use of a flow diagram | N/A |
| Descriptive data | 14 | (a) Give characteristics of study participants (eg demographic, clinical, social) and information on exposures and potential confounders | Results (P3)  Table 2 |
|  |  | (b) Indicate number of participants with missing data for each variable of interest | Methods (P10), Supplementary material 2 |
| Outcome data | 15 | Report numbers of outcome events or summary measures | Table 2 |
| Main results | 16 | (*a*) Give unadjusted estimates and, if applicable, confounder-adjusted estimates and their precision (eg, 95% confidence interval). Make clear which confounders were adjusted for and why they were included | Tables 3,4, and 5 |
|  |  | (*b*) Report category boundaries when continuous variables were categorized | Methods (P6) |
|  |  | (*c*) If relevant, consider translating estimates of relative risk into absolute risk for a meaningful time period | N/A |
| Other analyses | 17 | Report other analyses done—eg analyses of subgroups and interactions, and sensitivity analyses | Results (P5), Supplementary material 2 |
| Discussion | | |  |
| Key results | 18 | Summarise key results with reference to study objectives | Discussion (P1,3) |
| Limitations | 19 | Discuss limitations of the study, taking into account sources of potential bias or imprecision. Discuss both direction and magnitude of any potential bias | Discussion (P9-10) |
| Interpretation | 20 | Give a cautious overall interpretation of results considering objectives, limitations, multiplicity of analyses, results from similar studies, and other relevant evidence | Conclusion (P1-3) |
| Generalisability | 21 | Discuss the generalisability (external validity) of the study results | Discussion (P8) |
| Other information | | |  |
| Funding | 22 | Give the source of funding and the role of the funders for the present study and, if applicable, for the original study on which the present article is based | Funding information (P1) |

N/A – not applicable

**Source** www.strobe-statement.org.
