# Supplementary figures and images for: National policies and care provision in pregnancy and childbirth for twins in Eastern and Southern Africa: A mixed-methods multi-country study
Source: PLoS Med. 2019 Feb 19;16(2):e1002749. doi: 10.1371/journal.pmed.1002749 (PMC6380547; doi:10.1371/journal.pmed.1002749)

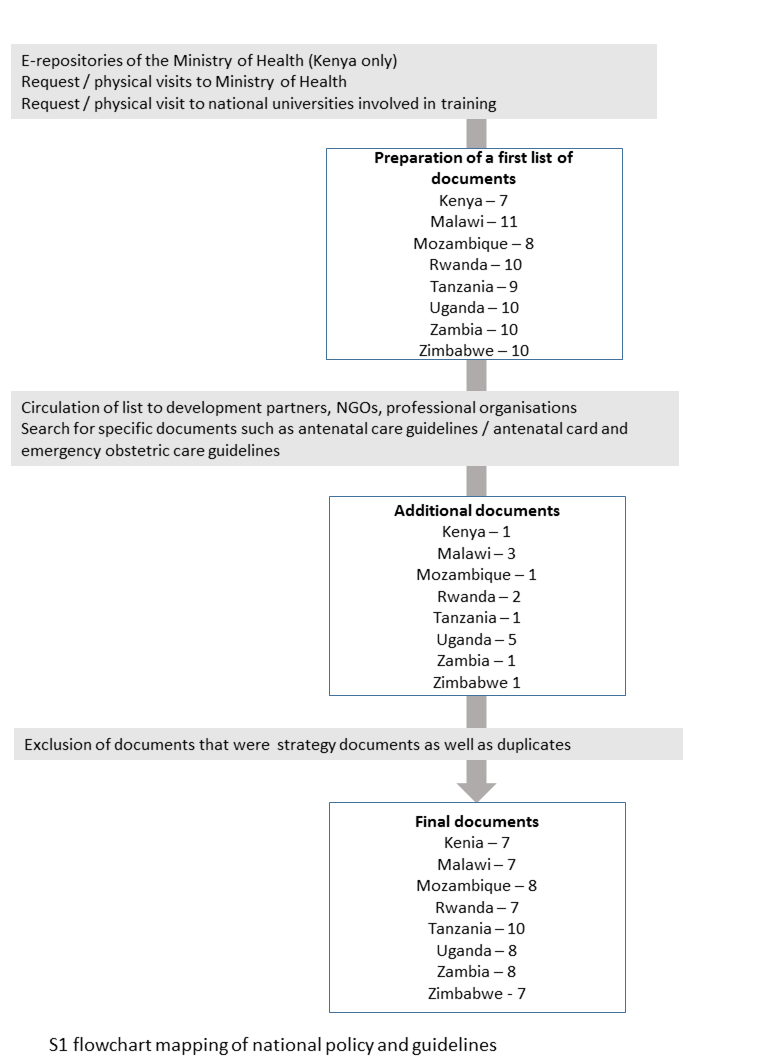

Supplement: S1 Fig — (TIF) [file pmed.1002749.s002.tif]
