# Supplementary material for: National policies and care provision in pregnancy and childbirth for twins in Eastern and Southern Africa: A mixed-methods multi-country study
Source: PLoS Med. 2019 Feb 19;16(2):e1002749. doi: 10.1371/journal.pmed.1002749 (PMC6380547; doi:10.1371/journal.pmed.1002749)
Supplement: S1 Text — (DOCX) [file pmed.1002749.s003.docx]

**S1 Text Sensitivity analysis of missing data in DHS**

Antenatal care

Percentage of women in analysis sample who had missing responses to content of ANC care (includes “Don’t know” and system missing “.” values)

|  | **Blood pressure measured at least once during ANC** | **Urine sample taken at least once during ANC** | **Blood sample taken at least once during ANC** | **Iron supplementation during pregnancy** | **Received information about complications during ANC** |
| --- | --- | --- | --- | --- | --- |
| Kenya | None | 0.03% | 0.03% | 0.28% | 0.11% |
| Malawi | None | None | None | 0.09% | not collected |
| Mozambique | None | None | None | 0.14% | 0.81% |
| Rwanda | 0.02% | 0.02% | 0.02% | 0.05% | 0.27% |
| Tanzania | None | None | None | 0.07% | not collected |
| Uganda | None | None | None | 0.11% | not collected |
| Zambia | None | 0.42% | 0.14% | 0.05% | 0.33% |
| Zimbabwe | None | None | None | 0.04% | not collected |

Delivery care

We used four variables, each with different decision in regard to how to deal with missing values.

1. Mode of delivery (vaginal or caesarean): No caesarean sections were reported in domestic environments (i.e. outside of health facilities). Deliveries where mode of delivery was missing were excluded from analysis of caesarean section.
2. Location of delivery: The small number of observations for which type of delivery facility was missing were categorised as lower-level facilities (i.e. not hospitals). This had an effect on two estimates: % of deliveries in any health facility and % in hospitals). The missingness in this variable was <1% in 4 countries and between 1% and 2% in the remaining four.
3. Newborn weighed: Women who had a missing value for whether their newborn was weighed were re-coded as Yes, the newborn was weighed. This is because weighing might occur while the newborn is taken away from the mother, reducing accuracy of her report. The extent of missingness was <1% in five countries, between 1% and 2% in further two countries, and 6.14% in one country (Malawi).
4. Early initiation of breastfeeding: We re-coded missing responses to the question on timing of initiation to “no” (did not initiate within an hour of birth). The extent of missingness ranged from 1.06% to 3.29% across the eight countries.

|  | % missing mode of delivery | % missing delivery location | % missing newborn weighed | % missing timing of breastfeeding initiation |
| --- | --- | --- | --- | --- |
| Kenya | 0.07% | 0.20% | 0.17% | 1.62% |
| Malawi | 0.29% | 1.35% | 6.14% | 2.38% |
| Mozambique | none | 1.31% | 0.34% | 3.29% |
| Rwanda | none | 0.02% | 0.35% | 1.06% |
| Tanzania | none | 1.46% | 1.09% | 1.62% |
| Uganda | 0.38% | 1.62% | 1.35% | 2.69% |
| Zambia | 0.03% | 0.02% | 0.32% | 2.39% |
| Zimbabwe | 0.06% | 0.00% | 0.05% | 2.36% |

Sensitivity analysis

We conducted sensitivity analysis for variables which exceeded 3% missingness.

Malawi - newborn weighed

| Current analysis (missing= weighed) | | | Missing= not weighed | | | Missing excluded from analysis | | |
| --- | --- | --- | --- | --- | --- | --- | --- | --- |
| Singleton | Twins | pvalue | Singleton | Twins | pvalue | Singleton | Twins | pvalue |
| 92.5% | 90.1% | 0.210 | 86.1% | 79.9% | 0.014 | 92.0% | 89.0% | 0.147 |

Mozambique - early initiation of breastfeeding

| Current analysis (missing= not early) | | | Missing= early | | | Missing excluded from analysis | | |
| --- | --- | --- | --- | --- | --- | --- | --- | --- |
| Singleton | Twins | pvalue | Singleton | Twins | pvalue | Singleton | Twins | pvalue |
| 75.9% | 67.4% | 0.038 | 78.70% | 75.70% | 0.453 | 78.10% | 73.50% | 0.280 |
