# Supplementary material for: National policies and care provision in pregnancy and childbirth for twins in Eastern and Southern Africa: A mixed-methods multi-country study
Source: PLoS Med. 2019 Feb 19;16(2):e1002749. doi: 10.1371/journal.pmed.1002749 (PMC6380547; doi:10.1371/journal.pmed.1002749)
Supplement: S1 Table — (DOCX) [file pmed.1002749.s004.docx]

**Links to the Ministries of Health to obtain the policies and guidelines and other organisations/agencies contacted to complete the search**

**Kenya**

Ministry of Health: <https://mail.health.go.ke/>

The Institute of Family Medicine, Chaka Complex, Musa Gitau Road, Off Waiyaki Way Nairobi, Kenya

University of Nairobi, Department of Obstetrics and Gynaecology, Kenyatta National Hospital, 2nd Floor Nairobi, Kenya

Jhpiego, Ring Road 14, Riverside, Nairobi, Kenya

Kenya Obstetrical and Gynaecological Society (KOGS), please add the office place, town country) KMA Center, Upper Hill 4th Floor Suite no: 407: Nairobi, Kenya

Moi University School of Medicine/Moi Teaching and Referral Hospital, Nandi Road, Eldoret ; Kenya

**Malawi:**

Ministry of Health:

<http://www.malawi.gov.mw/index.php?option=com_content&view=article&id=50&Itemid=22>

The Association of Obstricians and Gynaecologist of Malawi (AOGM), College of Medicine, Department of Obstetrics and Gynecologists, Private Bag 360, Chichiri, Blantyre, Malawi

University of Malawi. Dept of Gynaecology and Obstetrics, College of medicine, Private Bag 360, Chichiri Blantyre 3 Malawi

**Mozambique:**

Ministry of Health <http://www.misau.gov.mz/>

UNFPA office, Avenida Julius Nyerere 1419, Maputo

WHO office: Rua Joseph Kizerbo, 227. P.O. Box 377, Maputo, Maputo, Mozambique

Jhpiego office

Associacao Mocambican de Obstetras e Ginecologistas, Bairro Polana Cimento, Rua Do aloe vera 70 R/C, Maputo, Mozambique

Faculty of Medicine, Eduardo Mondlane University, Maputo, Mozambique. · Department of Gynecology and Obstetrics, Maputo Central Hospital, Maputo

**Rwanda:**

Ministry of Health: <http://www.moh.gov.rw/index.php?id=458>

Maternal, Child, and Community Health Division at the Rwanda Biomedical Center, KG 28 Ave #72 Kimihura, Box 4062 Kigali, Rwanda

In Rwanda all trainings, guidelines and all training materials have to be commissioned by the Ministry of Health why only one partner was contacted to obtain a complete list of all

**Tanzania:**

Ministry of Health: <http://www.moh.go.tz/en/contact-us>

Association of Gynaecologists and Obstetrician, AGOTA, P.O.Box 65117, Dar es Salaam, Tanzania

Tanzanian Midwives Association, TAMA, Kalenga street, 65524 Dar es Salaam

Muhimbili University of Health and Allied Sciences, P.O. Box 65001, Dar es Salaam

Jhpiego Tanzania, PO Box 9170, Plot No. 72, Block 45 B, Victoria Area, New Bagamoyo Road, Dar es Salaam, Tanzania

**Uganda:**

Ministry of Health: <http://www.health.go.ug/contact-us>

Association of Obstetricians and Gynecologists in Uganda, PO Box 11966, Kampala Uganda

Jhpiego, Uganda, Plot 36, Lower Naguru, East Road, Nakawa Divison, Kampala, Uganda

**Zambia**:

Ministry of Health: <http://www.moh.gov.zm/?page_id=5269>

Zambia Association of Gynaecologists & Obstetricians (ZAGO), C/o University Teaching Hospital, Department of OBS/GYN, P/Bag RW 1X, Lusaka, Zambia

University Teaching Hospital, University of Zambia, School of Medicine, P.O.BOX 50110, Lusaka, Zambia

University College of Nursing, P.O.BOX RW1, Lusaka, Zambia

Midwifery Association of Zambia C/O University Teaching Hospital, Lusaka Zambia

**Zimbabwe:**

Ministry of Health: [http://www.mohcc.gov.zw/#](http://www.mohcc.gov.zw/)

The Zimbabwe Society of Obstetricians and Gynaecologists, Gelfand Ritchken House, PO Box 3671, Harare, Zimbabwe

Dept of Obstetrics and Gynaecology, University of Zimbabwe College of Health Sciences, B-Floor, Old Health Sciences Building, P O Box A178 Avondale, Harare
