# Supplementary material for: National policies and care provision in pregnancy and childbirth for twins in Eastern and Southern Africa: A mixed-methods multi-country study
Source: PLoS Med. 2019 Feb 19;16(2):e1002749. doi: 10.1371/journal.pmed.1002749 (PMC6380547; doi:10.1371/journal.pmed.1002749)
Supplement: S2 Table — (DOCX) [file pmed.1002749.s005.docx]

**S2 Table Data extraction form**

| **Theme** | **What is mentioned**  **(examples)** | **Which document (with reference whether pre-service, in-service training material or guideline)** |
| --- | --- | --- |
| Twin / multiples mentioned as risk |  |  |
| Identification of twin pregnancies | Eg how to check what to do |  |
| Care during pregnancy | Any additional ANC visits recommended, any referral to hospital/ultrasound, any special investigation, birth preparedness, any mentioning of the high risk of preterm birth, etc …. |  |
| Advice on where to deliver | Do ANC guidelines give advice for hospital delivery / any referral? |  |
| Advice on operative delivery / Caesarean section |  |  |
| Advice on PNC | Any reference to the particular need of twins? |  |
| Breastfeeding | Do breastfeeding guidelines provide reference to how to manage to feed twins? What is recommend? |  |
| Kangaroo Mother Care | Same as above |  |
| Child health care | Same as above |  |
