# Supplementary material for: National policies and care provision in pregnancy and childbirth for twins in Eastern and Southern Africa: A mixed-methods multi-country study
Source: PLoS Med. 2019 Feb 19;16(2):e1002749. doi: 10.1371/journal.pmed.1002749 (PMC6380547; doi:10.1371/journal.pmed.1002749)
Supplement: S3 Table — (DOCX) [file pmed.1002749.s006.docx]

**S3 Table. Response options for facility-based delivery locations showing facility types categorised as hospitals, by country and sector, for surveys used**

|  | **PUBLIC SECTOR** | | **RELIGIOUS/ VOLUNTARY SECTOR** | | **PRIVATE SECTOR** | |
| --- | --- | --- | --- | --- | --- | --- |
| **Country** | **Hospital** | **Not hospital** | **Hospital** | **Not hospital** | **Hospital** | **Not hospital** |
| Kenya | Government hospital | Government health center, Government dispensary, Other public sector | Mission hospital/clinic |  | Private hospital/clinic | Nursing/maternity home, Other private sector |
| Malawi | Government hospital | Government health center, Government health post/outreach, Other public sector | CHAM/mission hospital | CHAM/mission health center | Private hospital /clinic | BLM, Other private sector |
| Mozambique | Public - central hospital, Public - provincial hospital, Public - rural hospital | Public - health center/post, Other public sector |  |  |  | Private clinic, Private medical consultant, Other private sector |
| Rwanda | Public referral hospital, Public provincial/ district hospital | Public health center, Public health post, Other public sector |  |  | Private - polyclinic | Private - clinic, Private - dispensary, Other private sector |
| Tanzania | National/zonal/ specialised hospital, Regional referral hospital, Regional hospital, District hospital | Health center, Government dispensary, Government clinic | Religious/voluntary: referral/specialist hospital, Religious/voluntary: district hospital, Religious/voluntary: Hospital | Religious/voluntary: Health center, Religious/voluntary: dispensary, Religious/voluntary: clinic | Private specialised hospital, Private hospital | Private health center, Private dispensary, Private clinic |
| Uganda | Government hospital | Government health center, Other public sector |  |  | Private hospital/clinic | Other private |
| Zambia | Government hospital | Government health center/post, Other public sector | Mission hospital/clinic |  | Private hospital/clinic | Other private sector |
| Zimbabwe | Public central hospital, Public provincial hospital, Public district hospital, Public rural hospital, Public urban municipal hospital | Public rural health center, Other public sector | Mission hospital/clinic |  | Private hospital/clinic | Other private medical sector |
