# Supplementary material for: National policies and care provision in pregnancy and childbirth for twins in Eastern and Southern Africa: A mixed-methods multi-country study
Source: PLoS Med. 2019 Feb 19;16(2):e1002749. doi: 10.1371/journal.pmed.1002749 (PMC6380547; doi:10.1371/journal.pmed.1002749)
Supplement: S5 Table — (DOCX) [file pmed.1002749.s008.docx]

**S5 Table International training programmes**

| **Theme** | **ALARM 22^nd^ edition 2015/2016** | | **ALSO Chapter G** | | **Life saving skills “Making it happen”**  **Chapter “Twin delivery” facilitators manual** | |
| --- | --- | --- | --- | --- | --- | --- |
|  | **What is mentioned** | **Where** | **What is mentioned** | **Where** | **What is mentioned** | **Where** |
| Identification of twin pregnancies | Detection of twin and chorionicity during routine first trimester ultrasound  Best early ultrasound 7-14 weeks | P9  “Delivery of twins” chapter, p6 | Scan  Larger uterus than dates, hyperemesis gravidarum, early pre-eclampsia, elevated maternal serum alpha fetoprotein levels, suggestive palpatory or auscultatory findings, polyhydramnios, ovulation induction, and family history | p19 | Can be discovered at abdominal palpation, scan, and abdominal and vaginal examination after the first baby is born | p 99 |
| Care during pregnancy | Make women aware on increased risk of preterm labour in twin delivery | “Delivery of twins” chapter, p12 | No preventive measures of bed rest, routine cerclage, and tocolytics can prevent premature birth  Contradicting recommendation of aspirin starting 10-12 weeks | p19 |  |  |
|  | Twin pregnancy is risk factor (p2)  Cervical length below 20mm is risk factor (p4) | “Preterm labour and preterm births” | Ultrasound every 4 weeks to detect discordant growth and placenta praevia  Fetal heart beat check at every ANC visit | p19 & 20 | - |  |
|  | Preterm PROM occurs in 17% of twin pregnancies (p1) | Prelabour Rupture of Membranes (PROM) |  |  | - |  |
| Advice on where to deliver |  |  |  |  | - |  |
| Care during delivery | Induction of labour for “Uncomplicated twin pregnancy ≥ 38 weeks” | “Induction of labour” chapter, p6 | Induction of labour in diamniotic/dichorionic twin week 38 and monoamniotic at week 36 to 37 | P20 | Check presentation, deliver per CS if transverse lie, check foetal heartbeat, check if augmentation with oxytocin is needed, after delivery of 1^st^ twin, put clamp on cord, do not deliver placenta until delivery of 2^nd^ twin,  External version to correct to longitudinal lie if membranes intact, if breech, rupture membranes and deliver as described for breech consider CS if not delivered after 2 hours | Slide 26, 27 and facilitator manual p 99/p100 |
|  | “verify that there is no twin” | “vaginal birth” chapter, p6 |  |  | Risk factor for PPH | Facilitator manual p100 |
|  | “increase in mortality”  “increase in antenatal care complication”  “Complications related to birth  -cord accidents, malpresentation, uterine atony, placental abruption, decrease in morbidity if birth interval > 30 minutes – vasa previa secondary – haemorrhage, postpartum depression | “Delivery of twins” chapter |  |  |  |  |
|  | VBAC outcomes are similar in singletons and twins | Vaginal Birth After Caesarean Section (VBAC) chapter |  |  |  |  |
| Advice on operative delivery / Caesarean section | “monochorionic, monoamniotic twins should be delivered 36-37 weeks per Caesarean section  Vaginal delivery if first twin cephalic presentation, consider augmentation of labour after the first twin is born | “Delivery of twins” chapter, p8 | Vaginal delivery if first twin in cephalic position  An experienced physician in breech delivery should be available for all twin deliveries  Delivery best in operation theatre to be able to shift quickly to Caesarean section | p21 | Advice on CS for 1^st^ twin if transverse lie, for 2^nd^ twin if not delivered after two hours or undeliverable breech | Facilitator manual |
|  | Be alert of PPH after twin delivery | “Delivery of twins” chapter, p13 |  |  |  |  |
| Advice on PNC |  |  |  |  | Not included |  |
| Breastfeeding |  |  |  |  | Not included (Facilitator manual only states: “Discuss aftercare for mother”) |  |
| KMC |  |  |  |  | Not included |  |
| Child health care |  |  |  |  | Not included |  |
| other | “When there is a surviving twin or a pregnancy immediately after a loss, the new baby’s identity may be confused with the idealized lost baby. The new baby may never live up to parents’ expectations and may be a focus of unresolved anger”. p5 |  |  |  |  |  |
