# Supplementary material for: National policies and care provision in pregnancy and childbirth for twins in Eastern and Southern Africa: A mixed-methods multi-country study
Source: PLoS Med. 2019 Feb 19;16(2):e1002749. doi: 10.1371/journal.pmed.1002749 (PMC6380547; doi:10.1371/journal.pmed.1002749)
Supplement: S6 Table — (DOCX) [file pmed.1002749.s009.docx]

S6 Table Deliveries resulting in live births in the survey recall period by number of birth and socio-economic status, by country

|  | | **Number of live birth deliveries** | | | **Household wealth score: mean (95% CI)** | | | **Maternal years of education attended: median; mean (95% CI)** | | |
| --- | --- | --- | --- | --- | --- | --- | --- | --- | --- | --- |
| Country | Survey | Singleton | Twins | Triplets and higher | Singleton | Twins | p-value | Singleton | Twins | p-value |
| Kenya | 2014 | 20,380 | 292 | 0 | 0.000290 (0.000282-0.000297) | 0.000301 (0.000278-0.000325) | 0.343 | 7; 7.6 (7.4-7.8) | 8; 8.1 (7.3-8.8) | 0.033 |
| Malawi | 2015-16 | 16,618 | 331 | 2 | 0.000278 (0.000271-0.000285) | 0.000270 (0.000248-0.000293) | 0.294 | 6; 5.5 (5.3-5.7) | 5; 5.2 (4.7-5.7) | 0.098 |
| Mozambique | 2011 | 10,657 | 221 | 1 | 0.000281 (0.000272-0.000291) | 0.000294 (0.000272-0.000315) | 0.663 | 3; 3.1 (2.9-3.3) | 2; 2.7 (2.2-3.2) | 0.079 |
| Rwanda | 2015 | 7,631 | 108 | 3 | 0.000282 (0.000275-0.000289) | 0.000239 (0.000212-0.000267) | 0.004 | 4; 4.4 (4.3-4.6) | 4; 4.2 (3.5-4.8) | 0.483 |
| Tanzania | 2015-6 | 9,865 | 181 | 2 | 0.000282 (0.000272-0.000292) | 0.000303 (0.000279-0.000328) | 0.136 | 7; 5.6 (5.4-5.8) | 7; 5.3 (4.6-6.0) | 0.511 |
| Uganda | 2016 | 15,012 | 252 | 2 | 0.000290 (0.000282-0.000299) | 0.000294 (0.000273-0.000314) | 0.994 | 6; 6.1 (5.9-6.3) | 6; 6.4 (5.8-7.1) | 0.302 |
| Zambia | 2013-4 | 13,034 | 207 | 3 | 0.000276 (0.000268-0.000284) | 0.000277 (0.000256-0.000299) | 0.897 | 6; 6.1 (5.9-6.2) | 6; 5.3 (4.7-5.9) | 0.029 |
| Zimbabwe | 2015 | 5,923 | 103 | 1 | 0.000291 (0.000280-0.000301) | 0.000327 (0.000301-0.000354) | 0.106 | 10; 9.0 (8.8-9.2) | 10; 9.4 (8.8-10.1) | 0.559 |
